# Formulation and evaluation of butenafine loaded PLGA-nanoparticulate laden chitosan nano gel

**DOI:** 10.1080/10717544.2021.1995078

**Published:** 2021-11-08

**Authors:** Sultan Alshehri, Syed Sarim Imam

**Affiliations:** Department of Pharmaceutics, College of Pharmacy, King Saud University, Riyadh, Saudi Arabia

**Keywords:** Butenafine, nanoparticles, irritation study, optimization, antifungal activity

## Abstract

The present research work is designed to prepare and optimize butenafine (BT) loaded poly lactic co glycolic acid (PLGA) nanoparticles (BT-NPs). BT-NPs were prepared by emulsification probe sonication method using PLGA (A), PVA (B) as polymer and stabilizer, respectively. The optimum composition of BT-NPs was selected based on the point prediction method given by the Box Behnken design software. The optimized composition of BT-NPop showed a particle size of 267.21 ± 3.54 nm with an entrapment efficiency of 72.43 ± 3.11%. The optimum composition of BT-NPop was further converted into gel formulation using chitosan as a natural polymer. The prepared topical gel formulation (BT-NPopG) was further evaluated for gel characterization, drug release, permeation study, irritation, and antifungal studies. The prepared BT-NPopG formulation showed optimum pH, viscosity, spreadability, and drug content. The release and permeation study results revealed slow BT release (42.76 ± 2.87%) with significantly enhanced permeation across the egg membrane. The irritation study data showed negligible irritation with a cumulative score of 0.33. The antifungal study results conclude higher activity than marketed as well as pure BT. The overall conclusion of the results revealed BT-NPopG as an ideal delivery system to treat topical fungal infection.

## Introduction

The topical delivery system is the most common route of administration for active therapeutics for the treatment of various skin diseases. The fungal infection can be caused by different micro-organisms like *Candida*, *Aspergillus*, and *Blastomyces.* There are different types of skin infections reported by these organisms. The infection caused by *candida* occurs between skin folds and where the heat and moisture lead to maceration as well as inflammation. In the case of Aspergillus, it mainly affects the person having low immunity. Aspergillosis infection found in few patients (5–10%) causes skin lesions. The lesions include single or multiple red or violet hardened plaques or papules. The key task for topical preparation is the penetration of the drug through the first layer of skin (stratum corneum) to reach the required therapeutic concentration to produce the pharmacological action (Mali et al., 2017; El-Housiny et al., 2018).

Various efforts have been reported to prepare suitable dosage regimens with improved physicochemical and biological characteristics to achieve a site-specific delivery (Sabir et al., 2019; Ameeduzzafar et al., 2021). Moreover, the nanoparticles are a novel delivery system that could improve the permeation as well as retention of drugs in the skin layers to treat deep fungal infections. These carriers have many other characteristics, such as sustained drug release, improve drug stability, reach the infected site, and increase therapeutic efficacy.

Butenafine hydrochloride (BT) is a new generation antimycotic drug with potent antifungal activity. It acts by inhibiting sterol synthesis and also blocks the squalene epoxidation (Ahmed et al., 2021). The chemical formula and molecular weight of BT are C_23_H_27_N.HCl and 353.93 g/mole (Rao et al., 2016). It is not administered by the oral route because it is highly metabolized by the liver and having low oral bioavailability (Mahdi et al., 2021). It is poorly soluble in water and soluble in methanol, chloroform, ethanol, and dichloromethane.

There are several synthetic, semi-synthetic, and natural polymers have been used to prepare the NPs. Among various polymers, PLGA is a commonly employed polymer due to its biodegradable and biocompatible characteristics. It is a copolymer of poly lactic acid), and poly (glycolic acid) and is available with different molecular weights (Ghasemian et al., 2013). PLGA is also used for the improvement of the pharmacokinetic and pharmacodynamic profile of many therapeutics, i.e. sildenafil citrate (Shahin et al., 2021), Tenofovir (Shailender et al., 2017), and zaleplon (Haggag et al., 2021). NPs prepared in the form of nanosuspension and having very low viscosity. So, it is difficult to apply to the skin so it may not reach the tissue site and give reduce efficacy. To overcome these limitations, the viscosity of prepared NPs is enhanced to retain the delivery system for maximum time on the skin.

There are many polymers like carbopol 934 (Said dos Santos et al., 2020; Batool et al., 2021), chitosan (Yenilmez et al., 2015; Dimitrovska et al., 2020; El Menshawe et al., 2020; Rabia et al., 2020), poloxamer (Chung et al., 2020; Cristiano et al., 2020) used for the formulation of topical gel. Among them, chitosan is a natural polymer used as a gelling agent for topical delivery. It is a biological macromolecular polymer prepared from the deacetylation of chitin (exoskeletons of crustaceans and insects). It is biodegradable, biocompatible, penetration enhancer, bioadhesive, and non-toxic with therapeutic properties like healing enhancer, antimicrobial, and hemostatic (Ameeduzzafar et al., 2014; Dave & Gor, 2018).

The present study aimed to prepare butenafine nanoparticles laden topical gel for topical application. The prepared BT-NPopG was evaluated for drug release, skin permeation and retention, gel characterization, and anti-fungal activity.

## Materials and methods

### Materials

Butenafine Hcl, Polyvinyl alcohol (PVA; 80–90% hydrolyzed, MW: 30,000–70,000), and Dialysis membrane MWCO-12000 was procured from Sigma Aldrich (USA). Poly lactic co glycolic acid (50:50) was procured from Evonik Industries. Chitosan (87% deacetylation) was obtained from sea India food, India. All the solvents and other ingredients used are of analytical grade.

### Methods

#### Development of PLGA nanoparticle

BT loaded PLGA nanoparticle was prepared by previously reported emulsification sonication method with slight modification (Khan et al., 2018). BT and PLGA (100–400 mg) were dissolved in organic solvent (5 ml, methanol: acetone 1:1). PVA in different concentrations (1–5%) was dissolved in water. The organic phase was added dropwise into the PVA aqueous solution for emulsification. Then the dispersion was sonicated (1–4 min) with the application of ultra-probe sonication (60 W/cm^3^, Hielscher, Ultra-sonics, Germany). The formulation was stirred at 1500 rpm for 6 h using a magnetic stirrer to evaporate the organic solvent. The prepared NPs were centrifuged at 15,000 rpm for 20 min at 4 °C (Remi, Mumbai, India). NPs were separated and lyophilized using cryoprotectant (mannitol 0.2%) and stored for further evaluation.

### Optimization

Box Behnken design (BBD; State-Ease Inc., Version 9, Minneapolis, USA) was applied to optimize the BT-NPs. The process of optimization performed using the independent variables PLGA (A, mg), PVA (B, %), and ST (C, min) has been used to optimize the NPs. The three independent variables were taken at three levels as low (−), medium (0), and high (+). Their effects were evaluated on PS (Y_1_) and EE (Y_2_) as dependent variables as shown in Table 1. The design showed a total number of fifteen formulations with three center points (same composition) were prepared and evaluated as depicted in Table 2. The data of dependent variables were fitted into the design model (linear, 2nd order, and quadratic model). The statistical regression analysis was also done to evaluate each response. Further, ANOVA was calculated to evaluate the best fit model. The polynomial equation and 3 D and contour plot were made to measure the effect of factors over the response.

**Table 1.** Optimization variables and responses used to optimize butenafine nanoparticles using Box-Behnken design software.

Factors	Levels used		
	Low level (−1)	Middle level (0)	High level (+1)
Formulation variables			
A: PLGA (mg)	100	250	400
B: PVA concentration (%)	1	3	5
C: Sonication time (min)	1	2.5	4
Responses			
Y_1_: Particle size (PS in nm)			
Y_2_: Entrapment efficiency (EE in %)			

**Table 2.** Formulation composition with their results used for optimization.

Formulation code	PLGA (mg)	PVA (%)	Sonication time (min)	Particle size (nm)	Entrapment efficiency (%)
	A	B	C	Y_1_	Y_2_
F1	100	1	2.5	222.78 ± 3.83	38.16 ± 1.11
F2	400	1	2.5	417.40 ± 5.23	76.51 ± 1.63
F3	100	5	2.5	217.59 ± 2.22	61.52 ± 2.54
F4	400	5	2.5	297.21 ± 2.45	82.49 ± 3.43
F5	100	3	1	213.12 ± 1.55	52.34 ± 3.32
F6	400	3	1	324.32 ± 2.03	77.94 ± 2.87
F7	100	3	4	150.08 ± 1.77	53.27 ± 1.97
F8	400	3	4	315.15 ± 2.66	87.04 ± 2.55
F9	250	1	1	319.13 ± 4.17	60.38 ± 3.21
F10	250	5	1	249.43 ± 3.43	74.99 ± 2.66
F11	250	1	4	276.56 ± 1.98	65.44 ± 2.21
F12	250	5	4	220.87 ± 2.65	80.16 ± 1.98
*F13	250	3	2.5	267.21 ± 3.54	72.43 ± 3.11
*F14	250	3	2.5	265.87 ± 3.11	71.23 ± 1.55
*F15	250	3	2.5	263.45 ± 2.65	71.87 ± 1.65

*Center point having same composition.

### Characterization

#### Particle characterization

Particle size (PS), polydispersity index (PDI), and zeta potential (ZP) of the prepared BT-NPs were measured by particle size analyzer (Malvern, UK). The prepared BT-NPs were diluted 100-fold and filled into a cuvette to analyze the PS and PDI. ZP is measured to assess the surface charge of the particle and the value ±30 mV is ideal for the stability of NPs. The sample is diluted and transferred to a cuvette (contain electrode) and placed into an instrument to analyze the result in triplicate (Xing et al., 2021).

### Drug entrapment and loading

The entrapment efficiency was analyzed by the indirect method using the ultracentrifugation process (Byeon et al., 2019). The prepared BT-NPs were filled into the centrifugation tube and run at 15,000 rpm for 30 min using a cooling centrifuge (Remi Mumbai, India). The supernatant was collected and measured the absorbance by UV-visible spectrophotometer (Shimadzu 1800, Kyoto, Japan) at 251 nm to calculate the BT content in each sample. EE (%) and DL (%) were calculated by the below equations (Din et al., 2017):

(1) $$\mathrm{EE}(\%)=\frac{BT\;\mathrm{content}\;\mathrm{added}-BT\;\mathrm{content}\;in\;\mathrm{supernatant}\;}{BT\;\mathrm{content}\;\mathrm{added}\;}\times 100$$

(2) $$\mathrm{DL}(\%)=\frac{BT\;\mathrm{content}\;\mathrm{added}-BT\;\mathrm{content}\;in\;\mathrm{supernatant}\;}{\mathrm{Weight}\;of\;\mathrm{NPs}\;}\times 100$$

### Scanning electron microscope

The high-resolution electron microscope (JEOL, JSM-SEm 5200, Tokyo Japan) was used to assess the surface morphology of the prepared BT-NPop. One drop of the sample was taken and spread over the slide and dried. The sample was evaluated at 15 kV voltage shadowed in a cathode evaporator with a gold layer. Finally, the sample was visualized under the microscope and the image was captured.

### Development of PLGA-NPs laden gel

Chitosan polymer was used to prepare BT-NPs laden gel. The weighed quantity of chitosan (10 mg/ml) was taken in a beaker containing acetic acid solution (1% w/v) and kept aside for overnight to complete solubilization. The formaldehyde (0.002 g/g of chitosan) and glycerin (0.2 g/g of chitosan) were added to the chitosan solution and stirred continuously until the formation of gel taken place. The optimized BT-NPs (1% of BT equivalent in gel) was incorporated in the above mixture and homogenized for uniform distribution of BT-NPs in the gel matrix. Finally, methylparaben (0.1% v/w) and triethanolamine were added as a preservative and to adjust pH (Yang et al., 2016). The gel was stored at ambient temperature for further evaluation.

### Characterization of BT-NPs laden gel (BT-NPopG)

The pH of BTNPopG was measured by a pH meter (Edge pH). The electrode of the pH meter was incorporated into the sample and allowed to be stable for few minutes. The reading of the test sample was noted from the pH meter in triplicate at room temperature (Ahmed et al., 2020). The viscosity of the prepared optimized formulation was evaluated by using a viscometer (Brook field viscometer, RV-2 T, USA) using spindle number 6 at room temperature. The extrudability of the prepared BT-NPopG was measured to check the extrusion of formulation from the tube after application of constant weight. The tube is filled with BT-NPopG (20 g) and a constant weight is applied from the crimp end. The cap of the tube opened and the extruded weight of gel was measured to find the extrudability (Kaur & Ajitha, 2019). The spreadability study was performed to evaluate the flow of semisolid formulations. BT-NPopG was performed using a glass plate. The glass slide was pre-marked and the test sample (1 g) was placed over the glass and the gel diameter was noted. Then the second glass was placed over the first plate and the weight (500 g) was applied over for 5 min and then the final diameter was noted. The spreadability was measured by the below equation (Gaba et al., 2015).

(3) $$\text{\% Spreadability}=\frac{\mathrm{Final}\;\mathrm{diameter}-\mathrm{Initial}\;\mathrm{diameter}}{\mathrm{Initial}\;\mathrm{diameter}}\times 100$$

### Drug content

BT-NPopG (100 mg) was taken and dissolved in the methanol. The sample was sonicated for 5 min and centrifuged at 10,000 rpm for 10 min. The supernatant was collected, filtered and BT content in the formulation was determined after suitable dilution in triplicate using a spectrophotometer (Pillai et al., 2015).

### Infrared spectroscopy

The drug-polymer interaction has been performed to evaluate the compatibility study. The samples pure BT, PVA, chitosan, physical mixture, and BT-NPopG have been evaluated to compare the spectral changes in the prepared formulations with pure samples. The study was performed by using infrared spectroscopy (ATR-FTIR, Bruker Alpha, Germany). The scanning was performed between 4000 and 400 cm^−1^.

### Drug release

The drug release study of BT-NPop and BT-NPopG was performed by dialysis bag. The samples containing 5 mg of BT were filled into a dialysis bag (MW 12,000 KDa) and both sides were tightly tied. The release media phosphate buffer (pH-6.8 with ethanol 20%) was taken in a beaker and placed over-temperature enabled magnetic stirrer at 37 ± 0.5 °C. Both the test samples were immersed into release media and stirred at 100 rpm. At a definite interval, 5 ml of released aliquots were collected and simultaneously replaced with the same volume of fresh media to maintain the study condition. The sample was filtered and analyzed in triplicate by a UV-visible spectrophotometer at 251 nm (Ahmed et al., 2021). The release data of BT-NPopG was fitted into different release kinetic models. The maximum regression value (*R*^2^) was taken into consideration for the selection of the best-fit release model and to determine the releases behavior.

### Permeation study

The *in-vitro* permeation study of BT-NPop and BT-NPopG was done by using the eggshell membrane. Egg membrane having a similar property to stratum corneum of human skin (Kunzi-Rapp et al., 1999; Saleem & Idris, 2016). The eggs used for the study were incubated at 37 °C to prepare the CAM. Cam was taken from the egg by making a hole at the blunt end of the egg. The CAM adhered to the eggshell was washed with the normal saline and then CAM was carefully removed. CAM was collected and further used for the permeation study (Tay et al., 2011). The membrane was placed between donor and acceptor compartment of diffusion cell having an effective area (3 cm^2^) and volume (20 mL). The permeation media phosphate buffer having pH 5.5 was filled into the acceptor compartment and temperature fixed at 37 ± 0.5 °C with continuous stirring (Pillai et al., 2015). The samples pure BT, BT-NPop, and BT-NPopG (∼5 mg of BT) were added to the donor compartment. The permeated sample (2 mL) was taken from the acceptor compartment at a fixed time and replaced with the same volume of fresh buffer. The samples were filtered, diluted, and analyzed at 251 nm. The permeation flux was calculated using the slope of the calibration plot by the below formula:

(4) $$ER=\frac{\left(\mathrm{Flux}\;of\;\mathrm{formulation}\right)}{\left(\mathrm{flux}\;of\;\mathrm{control}\right)}$$

The drug retention study was performed by collecting the tested membrane after the permeation study and washed to remove the excess of the formulation. The membrane was crushed and a specified amount of methanol was added to extract the BT from the membrane. The extract was collected, filtered, and further diluted to evaluate the BT content in the membrane.

### In-vitro irritation study

Hen egg chick chorioallantoic membrane (HET-CAM) was used to check the irritation of BT-NPopG Mehling et al., 2007). This method is commonly used because the capillary form in CAM is similar to the human skin membrane (Kunzi-Rapp et al., 1999). Hen eggs were collected from poultry form and incubated in a humidity chamber for 10 days at 37 ± 0.5 °C/55 ± 2% RH. The eggs were regularly manually rotated. On the 10th day, the eggs were taken out and the outer shell was carefully removed from the air chamber side. CAM was formed and BT-NPopG, normal saline (0.9% w/v, negative control), and NaOH (0.1 M, positive control) were added over CAM. The irritation potential was observed visually and a score was given as per the reference chart. The scoring of irritation was done as per the scale hemorrhage, lysis, and coagulation. The score was given as 0 (no reaction), to 3 (strong reaction). The following classification based on ≤0.8 (slightly irritating); >0.8 to <1.2 (moderately irritating); ≥1.2 to <2.0 (irritating); ≥2.0 severely irritating (Mehling et al., 2007).

### Antifungal activity

The antifungal efficacy of pure BT, marketed cream, and BT-NPopG was performed over the microorganisms in *Candida albicans* and *Aspergillus niger* using the cup plate method. The sabouraud dextrose agar nutrient media was prepared and sterilized at 121 °C. Each strain (0.1 mL) was added into a sterilized petriplate containing media (15 mL). The media was mixed properly and allowed for solidification. The well of 8 mm size was made with sterile borer and pure BT, BT-NPopG, and marketed cream (Derfina^®^ 1% w/w) were placed in the well and stand for 10 min. The plates were incubated into an incubator at 37 °C for 24 h. After that zone of inhibition (ZOI), each test sample was measured by a graduated scale at 24 and 48 h to compare the results.

### Stability study

The stability study was done as per ICH guidelines at different temperatures of 4 °C, 25 °C/60%RH, and 40 °C/75%RH. The sample was packed into a borosilicate glass vial and kept in a stability chamber (LIYI, LY-28, Dongguan, China) for 6 months. The gel was evaluated for physical appearance, viscosity, spreadability, and drug content as per a pre-determined time interval.

### Statistical analysis

The experiments were performed in triplicate to evaluate the results (mean ± *SD*). Graph pad prism was used to calculate the statistics. One-way ANOVA followed by the Tukey–Kramer multiple comparison tests was used to assess statistically significant. *p* < .05 was considered as statistical significance.

## Result and discussion

### Optimization

**Figure 1.**
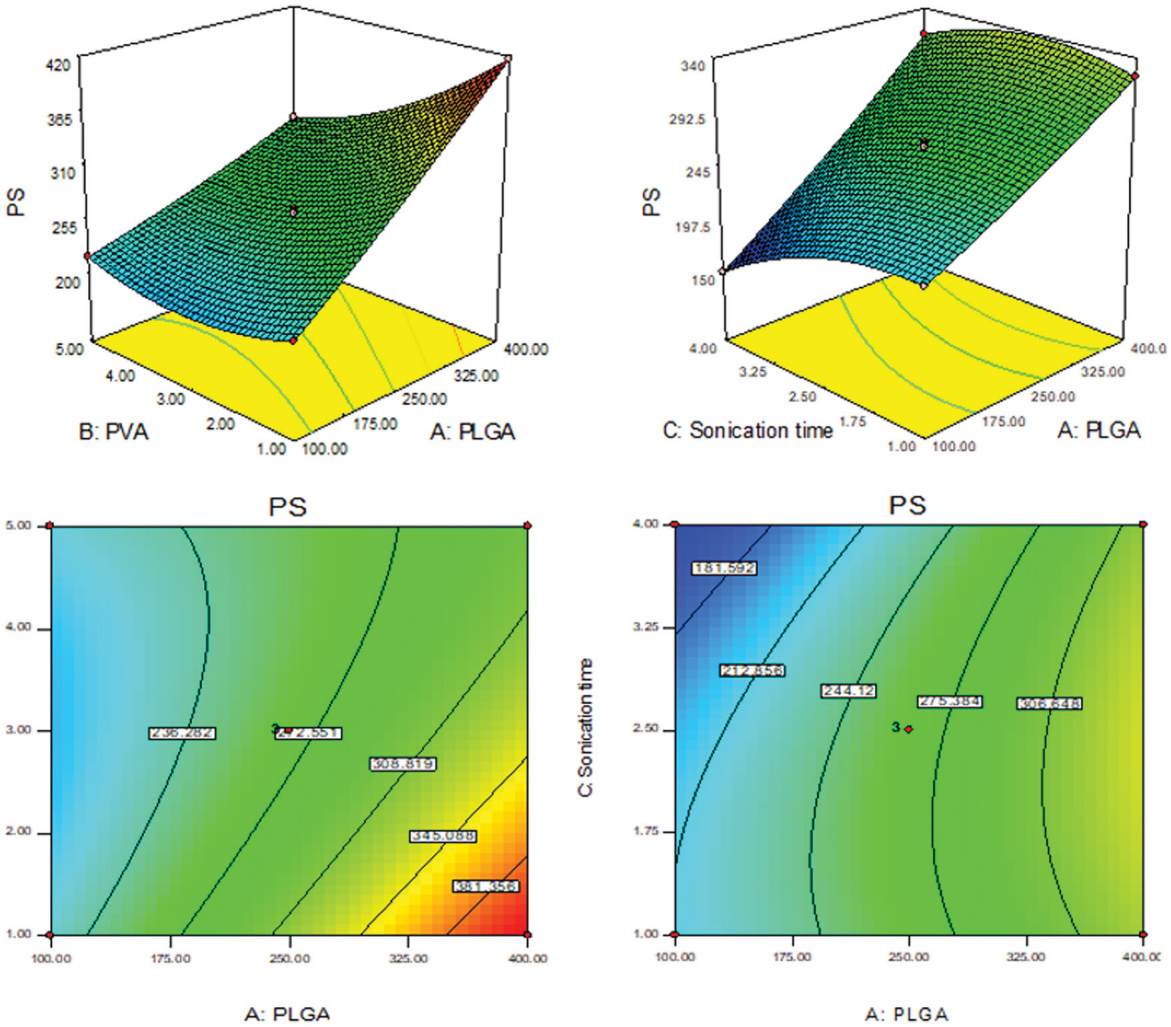
Effect of formulation independent variables [(PLGA (A), PVA (B), ST (C)] on particle size (Y_1_).

**Figure 2.**
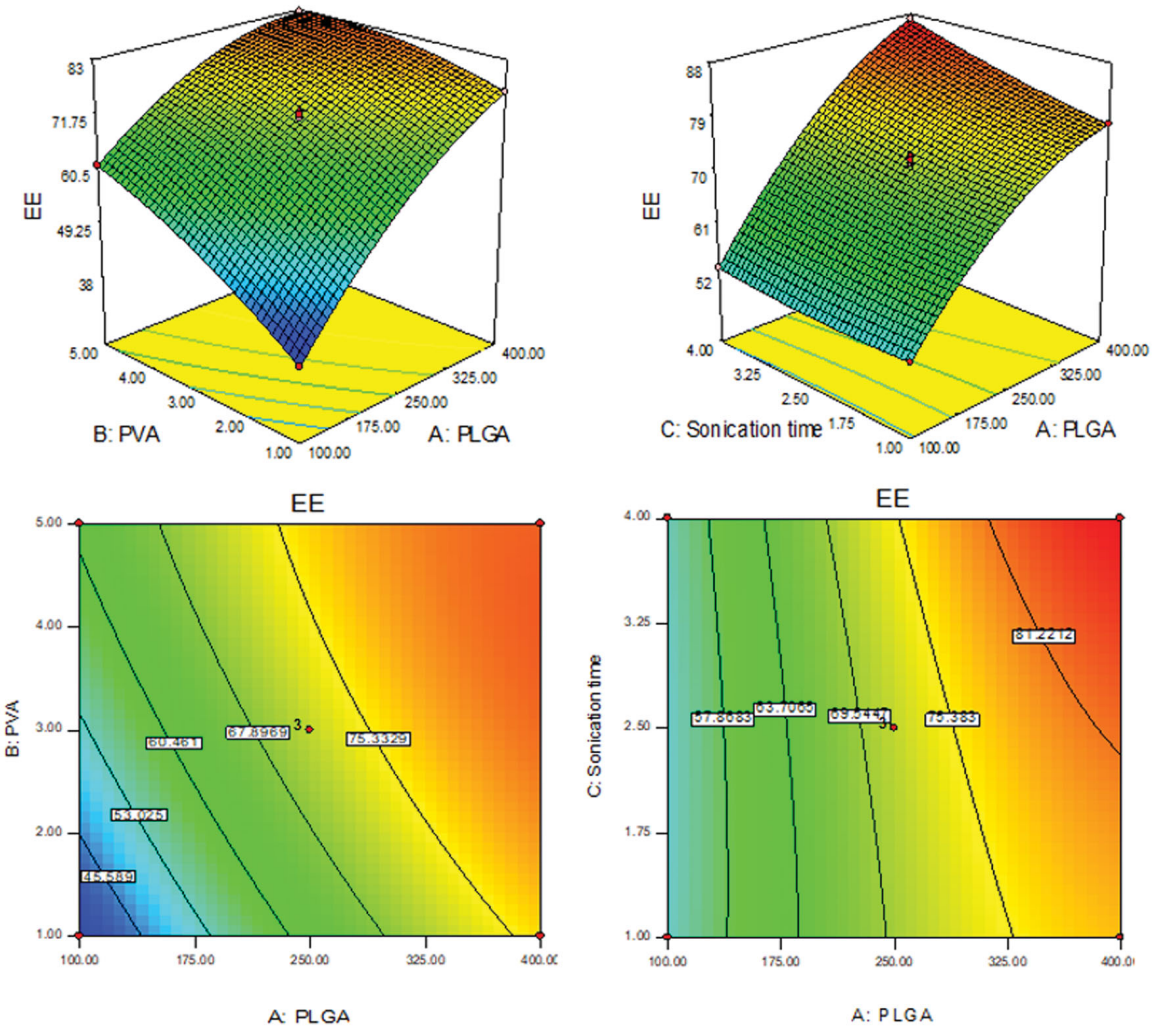
Effect of formulation independent variables [(PLGA (A), PVA (B), ST (C)] on encapsulation efficiency (Y_2_).

**Figure 3.**
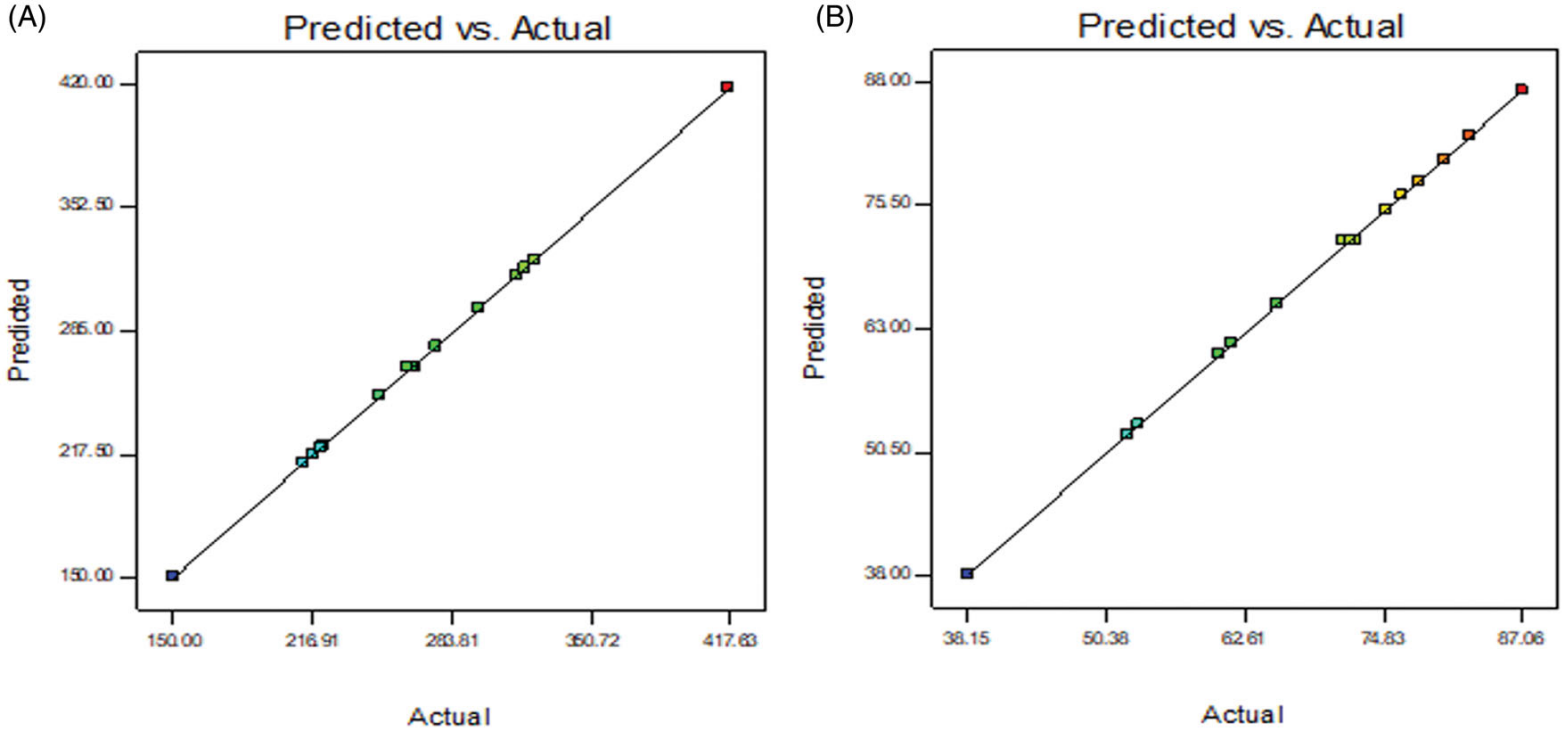
Actual and predicted value of (A) particle size as Y_1_ and (B) encapsulation efficiency as Y_2_.

BT-NPs were prepared by emulsification sonication method and further statistically optimized by Box Behnken design. The design depicted 15 formulation runs and their results are shown in Table 2. There was a significant difference in the PS and EE was observed between prepared BT-NPs. The value of each response [(PS (Y_1_) and EE (Y_2_)] fitted into different design models and the result showed quadratic model as best fit model because of its maximum regression value than others model (Tables 3 and 4). The lack of fit of each model for both responses was evaluated and found that the model was well fitted. The quadratic model of each response was further verified by ANOVA and the sum of square, degree of freedom, *F*-value, and *p*-value of each factor for each response was also calculated. Further, the effect of an individual as well as combined independent variables over each response was explained by the 3D-surface plot (Figures 1 and 2). The closeness between the actual and predicted value of each response of all formulations (F1–F15) was depicted in Figure 3. The points of all factors were found linearly very close to each other, so from that graph, we can say that the findings of the study are well accepted with the method used.

**Table 3.** Statistical summary of all applied models for response particle size (Y_1_).

Source	Sum of squares	*df*	Mean square	*F* value	*p*-Value	Remark
*Sequential model sum of squares*						
Mean *vs.* total	1,076,288	1	1,076,288	–	–	–
Linear *vs.* mean	48,266.92	3	16,088.97	25.0	<.0001	–
2FI *vs.* linear	4084.25	3	1361.42	3.64	.06	–
Quadratic *vs.* 2FI	2982.98	3	994.33	586.3808	<.0001	Suggested
*Lack of fit tests*						
Linear	7067.71	9	785.30	196.32	.0051	–
2FI	2983.46	6	497.24	124.31	.0080	–
Quadratic	0.48	3	0.16	0.04	.9866	Suggested
Source	Std. Dev.	*R* ^2^	Adjusted *R*^2^	Predicted *R*^2^	PRESS	Remark
*Model summary statistics*						
Linear	25.36	0.8721	0.8372	0.7267	15,120.77	–
2FI	19.33	0.9459	0.9054	0.7146	15,794.45	–
Quadratic	1.30	0.9998	0.9995	0.9995	25.65	Suggested

**Table 4.** Statistical summary of all applied models for response entrapment efficiency (Y_2_).

Source	Sum of squares	*df*	Mean square	*F* value	*p*-Value	Remark
*Sequential model sum of squares*						
Mean *vs.* total	70,141.98	1	70,141.98	–	–	–
Linear *vs.* mean	2242.61	3	747.54	41.10	<.0001	–
2FI *vs.* linear	91.98	3	30.66	2.27	.1574	–
Quadratic *vs.* 2FI	107.32	3	35.77	246.6	<.0001	Suggested
*Lack of fit*						
Linear	199.31	9	22.14581	61.42514	.0161	–
2FI	107.32	6	17.88802	49.61545	.0199	–
Quadratic	0.004	3	0.001365	0.003787	.9996	Suggested
Source	Std. Dev.	*R* ^2^	Adjusted *R*^2^	Predicted *R*^2^	PRESS	Remark
*Model summary statistics*						
Linear	4.26	0.9181	0.8957	0.8395	391.96	–
2FI	3.67	0.9557	0.9226	0.8271	422.27	–
Quadratic	0.38	0.9997	0.9991	0.9993	1.68	Suggested

### Effect of formulation factors (A, B, C) over PS

The PS of BT-NPs was found between 150.08 nm (F7) and 417.40 nm (F2). The result showed a significant difference in the PS. The difference in the size was found due to the variation in the formulation composition. The formulation (F2) having the composition PLGA concentration (100 mg), PVA concentration (3% w/v), and ST (4 min) showed the lowest size. The formulation (F7) having PLGA concentration (400 mg), PVA concentration (1% w/v), and ST (2.5 min) has shown the largest size. The polynomial equation explained the influence of formulation variables over the particle size individually (A, B, and C) and in combination (AB, AC, and BC). It was observed that increasing the PLGA concentration (A) the particle size increases due to an increase in viscosity of the organic phase. Due to improper stabilization and reduced delamination of ultrasonic waves the particle size increases. Similar results were observed by previously reported work (Shailender et al., 2017). The second variable, the increase in PVA concentration (B) showed dual behavior. The initial increase in PVA concentration from 1 to 2% leads to a decrease in size. The further increase in PVA concentration gives a slight increase in size. The optimum concentration can reduce the interfacial tension between aqueous and organic phases and lead to reduced particle size. Moreover, an increase the sonication time (C) leads to a decrease in the particle size due to the high energy supply which leads to the breakdown of a particle (Mainardes & Evangelista, 2005). The finding of the study was further interpreted mathematically by using the polynomial equation:

(5) $$\begin{array}{l} \text{Particle size }\left(\text{PS},{\text{ Y}}_{\text{1}}\right)=+\text{265}.00+\text{68}.\text{78 A}\\ -\text{31}.\text{34 B}-\text{17}.\text{89 C}-\text{28}.\text{75AB}+\text{13}.\text{5}0\text{ AC}\\ +\text{3}.\text{5}0\text{ BC}+\text{3}.{\text{88 A}}^{\text{2}}+\text{19}.{\text{88 B}}^{\text{2}}-\text{18}.{\text{38 C}}^{\text{2}}. \end{array}$$

Where A, B, and C are the model terms, negative and positive signs represent the synergistic and antagonistic effect on response. A, B, C, AB, AC, BC, A^2^, B^2^, and C^2^ are significant model terms because the *p*-value <.05, it means all model terms significantly affect particle size. The model *F*-value is 3625.78 denotes the model is significant. The lack of fit is insignificant (*p* = .98) indicated it is well fitted the model. The predicted *R*^2^ of 0.9995 is closer to the Adjusted *R*^2^ of 0.9996. Adequate precision of 251.4 designates an adequate signal.

### Effect of formulation factors (A, B, C) over EE

EE (Y_2_) of the prepared BT-NPs were found between 38.16 ± 1.11% (F1) − 87.04 ± 2.55% (F8). From the results, it can be observed that there was a significant difference in the encapsulation efficiency. The difference in BT encapsulation was due to the variation in the formulation compositions. The formulation (F1) showed the lowest encapsulation efficiency with the formulation composition PLGA concentration (400 mg), PVA concentration (5% w/v), and ST (2.5 min). The maximum encapsulation efficiency observed with the formulation (F4) PLGA concentration (400 mg), PVA concentration (1% w/v), and sonication time (2.5 min) has shown the largest size. The polynomial equation is used to explain the influence of formulation variables over the encapsulation efficiency individually (A, B, and C) as well as combinedly (AB, AC, and BC). It supports the quadratic effect of all responses over the encapsulation efficiency. The first variable PLGA concentration (A) showed a positive effect on the encapsulation efficiency. As the concentration of PLGA increases from 100 to 400 mg, the entrapment efficiency also increases due to the high viscosity of the organic phase leads to less partitioning between the aqueous and organic phases. It helps to reduce the amount of drug diffuses to the aqueous phase. A similar type of result was reported by previously reported work (Shailender et al., 2017). In the case of the second variable PVA (B) also showed dual behavior on encapsulation efficiency. With an increase in the PVA concentration from 1 to 3% w/v, the entrapment efficiency increases. With a further increase in PVA concentration up to 5% w/v the encapsulation efficiency slightly decreases. The decrease in encapsulation efficiency may be due to the hydrophobic property of drug molecules in the particles. With the increase in PVA concentration in the external phase, BT may diffuse out from the particles and solubilize as micelles in the aqueous phase (Sharma et al., 2016). The maximum amount of BT solubilizes and is entrapped to the PLGA as well as helps to less amount of drug diffusion to an aqueous phase. Moreover, as the sonication time (C) increases, the entrapment efficiency decreases because there may be leaching of the drug takes place due to a high-energy supply (Mainardes & Evangelista, 2005). The effect of independent variables is also explained by the mathematical relationship of independent variables on the encapsulation efficiency by the polynomial equation:

(6) $$\begin{array}{l} \text{Entrapment efficiency }\left(\text{EE},{\text{ Y}}_{\text{2}}\right)=+\text{ 71}.\text{84 }+\text{ 14}.\text{84 A }\\ +\text{ 7}.\text{33 B }-\text{ 2}.\text{53C }-\text{ 4}.\text{34 AB }+\text{ 2}.0\text{4 AC}\\ \;+\;0.0\text{28 BC }-\text{ 4}.{\text{89 A}}^{\text{2}}-\text{ 2}.{\text{29 B}}^{\text{2}}+\;0.{\text{69 C}}^{\text{2}}. \end{array}$$

where A, B, and C are the model terms, negative and positive signs represent the synergistic and antagonistic effect on response. A, B, C, AB, AC, BC, A^2^, B^2^, and C^2^ are significant model terms because the *p*-value <.05, it means all model terms significantly affect entrapment efficiency. The model *F*-value is 1870.78 denotes the model is significant. The lack of fit is insignificant (*p* = .9996) indicated it is well fitted the model. The Predicted *R*^2^ of 0.9993 is in close agreement with the Adjusted *R*^2^ of 0.9992. Adequate precision of 157.28 designates an adequate signal.

### Point prediction

**Figure 4.**
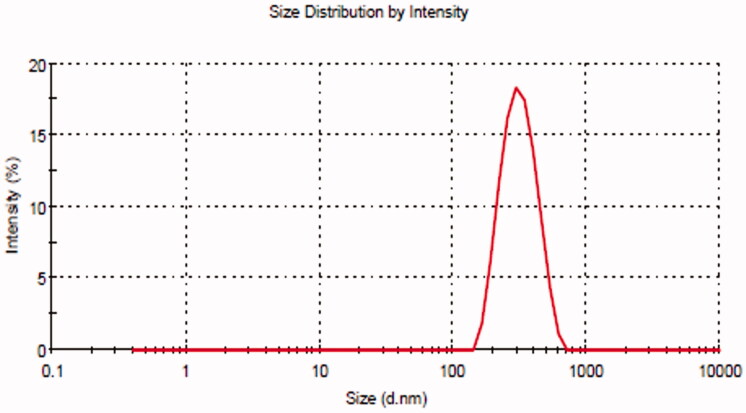
Particle size of the optimized butenafine nanoparticle (BT-NPop).

**Figure 5.**
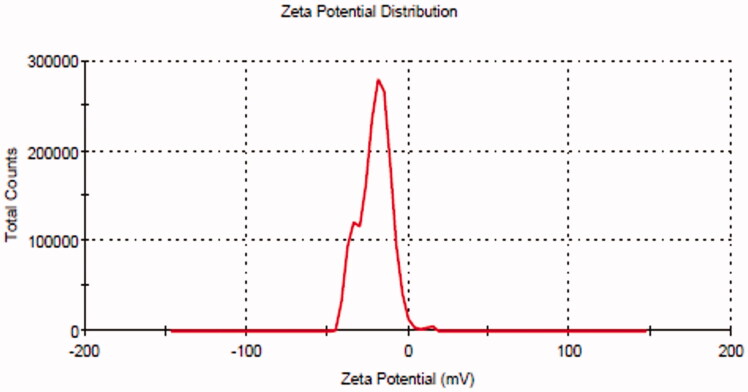
Zeta potential value of the optimized butenafine nanoparticle (BT-NPop).

The selection of optimized BT-NPs from the used different compositions was performed by the point prediction method of the software. The actual and predicted value showed close relation in the results which is also shown graphically in Figure 3. This result can also support the used method is ideal for the preparation of NPs. The desirability value of each factor was calculated from the software and the result showed the value between 0.988 and 0.993, i.e. (closer to 1) which confirms that the method is robust. The selection was based on the minimum particle size and maximum entrapment efficiency. The composition of optimized BTNPs (denoted as BT-NPop) having PLGA concentration (250 mg), PVA concentration (3% w/v), and sonication time (2.5 min). The above composition showed the actual particle size of 267.21 ± 3.54 nm (Figure 4), PDI of 0.227, and entrapment efficiency of 72.43 ± 3.11%. The software showed a predicted particle size of 265.02 nm with an entrapment efficiency of 71.84%. BT-NPop showed a negative zeta potential value (−20.3 mV, Figure 5). The stable NPs must have a ZP value between −30 and +30 mV. The high and low values from the reference lead to instability and aggregation. The optimized formulation BT-NPop was also evaluated for the drug load and the value was found to be 16.34 ± 0.76%.

### Scanning electron microscope

**Figure 6.**
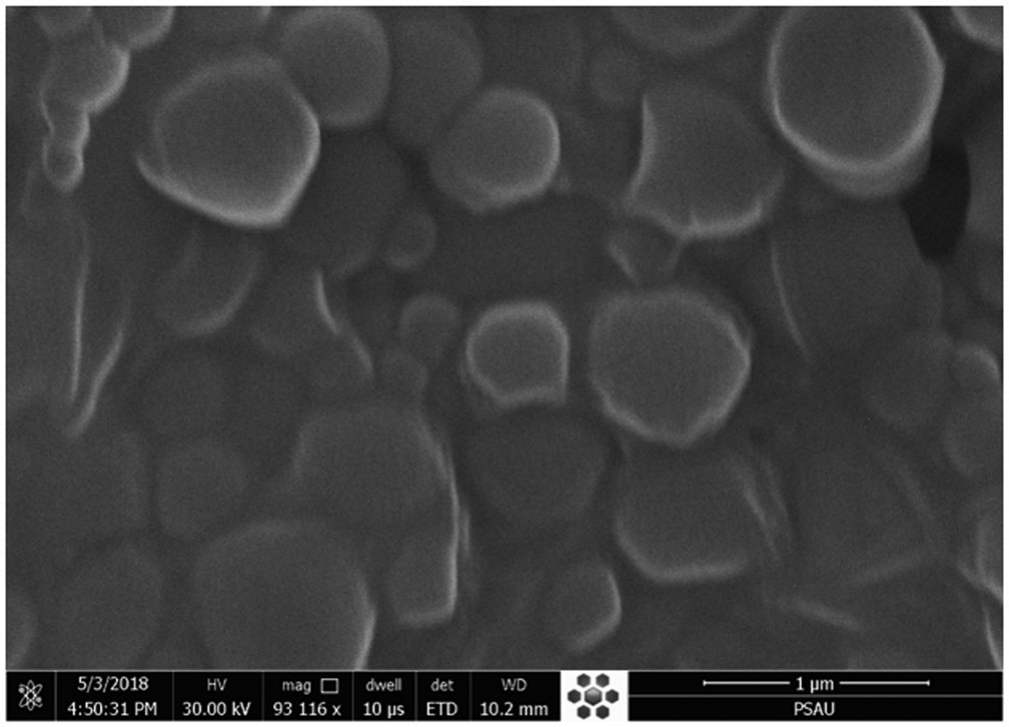
Scanning electron microscope image of optimized butenafine nanoparticle (BT-NPop).

The prepared BT-NPop has been evaluated for surface morphology using a scanning electron microscope (SEM). The image showed scattered particles with a smooth surface (Figure 6). There was no aggregation of particles observed. There was no significant difference in particle size was observed.

### Development of BT-NPopG

BT-NPop was successfully dispersed into chitosan polymer gel (10 mg/mL) and prepared BT-NPopG containing 1% of BT. The gelling agent was optimized by taking different concentrations (5, 10, and 15 mg) of chitosan to select the optimum concentration. The gelling agent at (10 mg/mL) concentration showed an ideal spreadability value than different formulations.

### Characterization of gel

The pH of BT-NPopG was found to be 6.6 ± 0.3, which is ideal for the topical formulation and does not produce any irritation (Riaz et al., 2020). The viscosity was also assessed to check the flow property of the prepared gel and the value was found to be 35,276 ± 14 cps. The viscosity lies between 35,000 and 40,000 cps is ideal for topical preparation, which gives good flow property and spreadability (Ahmed et al., 2021). The optimum viscosity value gives better adherence to the skin and spreadability. The viscosity of the gel also depends on the particle size and PDI value. The larger particle size gives higher viscous gel (Koca et al., 2018). The spreadability and extrudability of BT-NPopG were measured and the result showed 10.76 ± 1.09 cm and 14.98 ± 1.78 g/cm^2^, respectively. A good spreadability and extrudability value were found due to the optimum viscosity of the gel. The optimum viscosity and spreadability of topical gel can easily be applied to the skin in the form of a thin layer (Motawea et al., 2018).

### Drug content

The drug content of the prepared BT-NPopG was found to be 99.11 ± 1.14%. It represents that the maximum amount of BT dispersed uniformly in the gel matrix and it is in a desirable character of the prepared formulations. The high drug content supports the authenticity of the used method to prepare gel formulations.

### Differential scanning calorimetry

**Figure 7.**
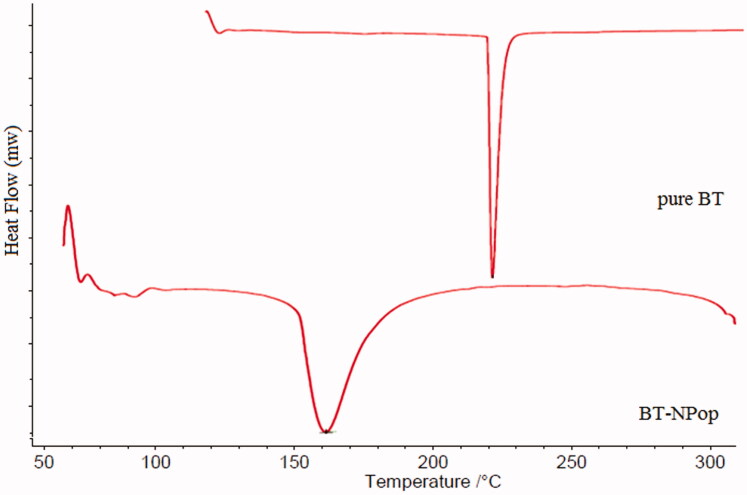
DSC image of pure butenafine and optimized butenafine nanoparticle (BT-NPop).

DSC study of pure BT and BT-NPopt was performed and thermograms were used to check the change in endothermic peak (Figure 7). The pure BT showed the endothermic peak at 221.5 °C, corresponding to its melting temperature. The thermogram of BT-NPop showed no peak of BT at the melting temperature of BT. The disappearance of the BT peak showed the uniform distribution and solubilization of BT in the polymer. The other peaks in the BT-NPop may be of the used carriers.

### Infrared spectroscopy (FT-IR)

**Figure 8.**
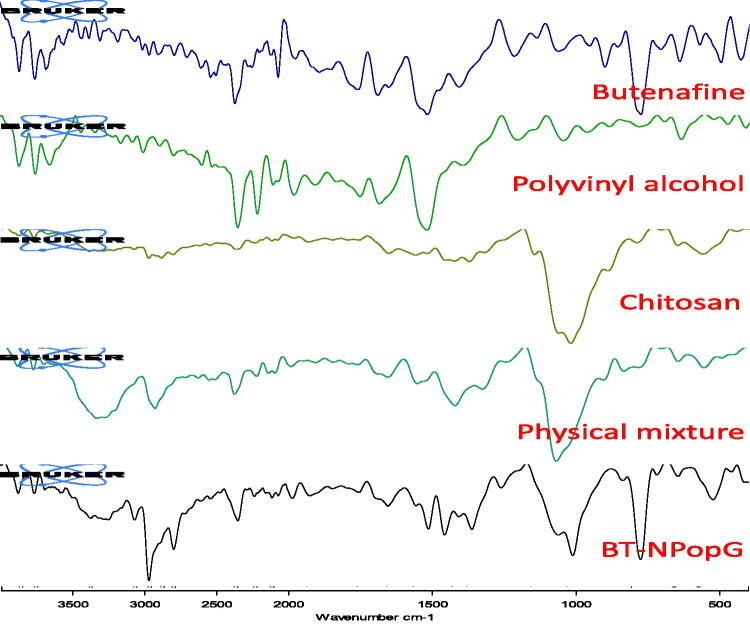
Infrared spectroscopy of pure butenafine, polyvinyl alcohol, chitosan, physical mixture, and optimized butenafine nanoparticle gel (BT-NPopG).

FTIR study of the pure BT, chitosan, polyvinyl alcohol, physical mixture, and BT-NPopG was performed to check conformational changes in the characteristic peaks (Figure 8). The pure BT showed a sharp stretching CH_2_ peak at 2955.74 cm^−1^, C–CH_3_ peaks at 2892.39 cm^−1^, C=C aromatic peak at 1650.84 cm^−1^, and C–N stretching peaks at 1216.77 cm^−1^, respectively. The carrier chitosan exhibited –OH stretching peak at 3366 cm^−1^, C–H stretching peak at 2955.53 cm^−1^, and CH_2_-OH stretching peak at 1372.30 cm^−1^. The second carrier, polyvinyl alcohol (PVA) exhibited free-OH stretching vibration at 3743.40 cm^−1^. The ester (R-COO-R′) group of PVA depicted stretching peaks at 1754.39 cm^−1^. The physical mixture exhibited the CH2 stretching peaks of the pure BT with a slight deviation at 2905.08 cm^−1^. The aromatic C = C and C–N stretching peaks were also observed at 1651.48 and 1231.61 cm^−1^. BT-NPopG showed the CH_2_ stretching peaks of BT with slight changes at 2952.43 cm^−1^. The other peaks of BT were also observed for C–CH_3_, aromatic C = C, and C–N peaks at 2781.67, 1652.04, and 1261.32 cm^−1^, respectively. The carrier peak for alcoholic chitosan was observed at 3353.42 cm^−1^, which exhibited a slight deviation from the original carrier peak. The ester peak of PVA was also observed with a slight deviation at 1751.39 cm^−1^. The carrier’s other spectral peaks were also observed in the physical mixture and BT-NpopG.

### Drug release study

**Figure 9.**
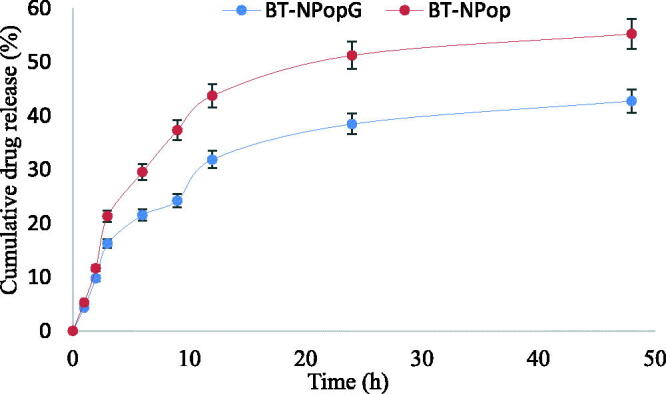
Comparative drug release profile of butenafine nanoparticle (BT-NPop) and butenafine nanoparticles laden gel (BT-NPopG). The study was performed in triplicate and data was shown as mean ± *SD*.

The comparative drug release study was performed to evaluate the amount of BT releases in the tested time. The results showed significant differences in the release profile of BT NPop, and BT-NPopG (Figure 9). BT-NPop and BT-NPopG showed 55.23 ± 4.55 and 42.76 ± 2.87% BT release in the 48 h study. The release pattern was found to be sustained. There was lesser amount of BT release (10%) in the first 2 h and at a later stage, the release was slowly increased. PLGA has been reported for the slow drug release which is ideal for the topical formulation. Due to the slow drug release, the maximum drug concentration reaches the target site. BT-NPopG also depicted a significantly slower BT release than BT-NPop. The reason for the slow BT release from the gel formulation is due to the slow diffusion of BT from the chitosan gel matrix. From the gel, the drug first releases from the PLGA then it will diffuse from the chitosan so the drug release was found to be slower. The release data of BT-NPopG was fitted into various release models, i.e. zero-order (0.8108), first-order (0.8984), Higuchi (0.9428), and Korsmeyer-Peppas model (0.9222). The regression coefficient was maximum for the Higuchi model (*R*^2^ = 0.9428), so considered it is the best-fit release kinetic model.

### Permeation and retention study

**Figure 10.**
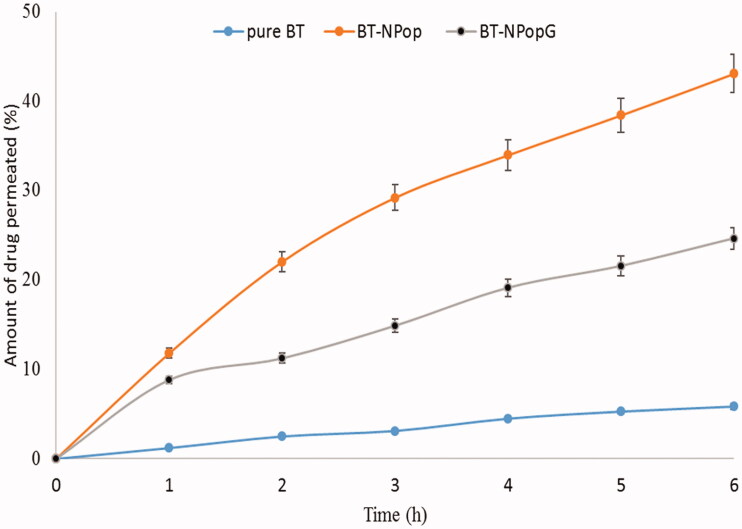
Comparative permeation profile of pure butenafine, butenafine nanoparticle (BT-NPop), and butenafine nanoparticles laden gel (BT-NPopG). The study was performed in triplicate and data was shown as mean ± *SD*.

The permeation study was performed to evaluate the permeation of BT from BT-NPopG and BT-NPop using an eggshell membrane. The study results revealed a significant difference in the permeation flux value among both these formulations (Figure 10). The amount of BT permeated from pure BT, BT-NPop, and BT-NPopG was found to be 17.56 ± 1.11 µg/cm^2^/h (105.36 ± 4.89 µg), 79.12 ± 9.23 µg/cm^2^/h (474.72 ± 21.78 µg) and 137.11 ± 10.54 µg/cm^2^/h (822.11 ± 37.23 µg). The enhancement ratio was found to be 4.5-fold and 7.8 from BT-NPop and BT-NPopG, respectively. There was a significant (*p* < .01) difference in the flux value was observed due to the presence of chitosan in the BT-NPopG. Chitosan has the property of mucoadhesion, disruption, and/or modulation of the tight junction of mucosa and reduces the strength of tight junction (Yeh et al., 2011). The presence of a positive charge on chitosan molecules also helps to enhance the permeation. It can interact with the negatively charged sialic acid on the cell membranes gives an opening to the tight junction and promotes drug permeation (Casettari & Illum, 2014). The total amount of BT retained in the membrane was also calculated and the pure BT, BT-NPop, and BT-NPopG showed 314.64 ± 12.43 µg, 889.11 ± 28.56 µg/cm^2^/h, and 1237.56 ± 31.66 µg. There is a higher amount (∼3-fold) of BT retained in the membrane make it an ideal delivery system for topical delivery.

### In-vitro irritation study

HET–CAM method was used to evaluate the irritation study of the prepared BT-NPopG using egg membrane and irritation score was calculated (Table 5). The samples were tested for lysis, hemorrhage, and coagulation for 5 min. The negative control (0.9% w/v, sodium chloride) exhibited zero scores (no hemorrhage, no damage to blood capillary), so considered as non-irritant. The positive control (0.1 M NaOH) exhibited a cumulative score of 7.33 (>2). The high score indicates severe irritants to the tissue. The prepared BT-NPopG treated CAM also showed a cumulative score of 0.33 with no sign of lysis, hemorrhage, and coagulation. The overall study design revealed a score between 0 and 0.9 was reported as a non-irritant and safe.

**Table 5.** HET-CAM irritation score treated with different groups.

Test sample	Egg	Time (min)				Overall score
		0	0.5	2	5	
BT-NpopG	Egg 1	0	0	1	0	0.33
	Egg 2	0	0	0	0	
	Egg 3	0	0	0	0	
	Mean score	0	0	0.33	0	
0.1 M NaOH (positive control)	Egg 1	0	2	2	3	7.33
	Egg 2	0	1	3	3	
	Egg 3	0	2	3	3	
	Mean score	0	1.66	2.67	3	
Normal saline (0.9%) negative control	Egg 1	0	0	0	0	0
	Egg 2	0	0	0	0	
	Egg 3	0	0	0	0	
	Mean score	0	0	0	0	

### Antifungal efficacy

**Figure 11.**
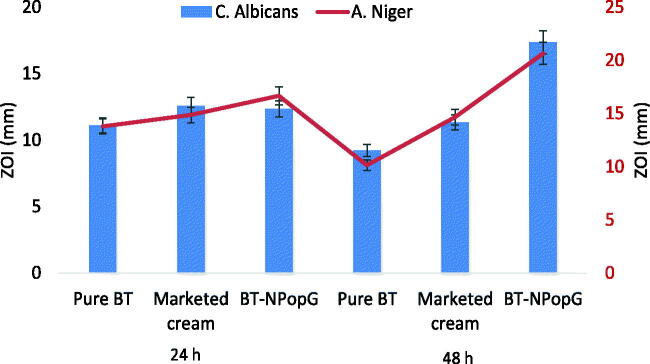
*In vitro* antibacterial activity of the pure butenafine, marketed cream, and BT-NPopG. The study was performed in triplicate and data was shown as mean ± *SD*.

Figure 11 expressed the comparative antifungal activity of pure BT, marketed cream, and BT-NPopG. The pure BT, marketed cream, and BT-NPopG showed the zone of inhibition 11.1 ± 1, 12.58 ± 1.5, and 12.35 ± 1.25 mm against *C. albicans* at 24 h. At 48 h, the pure BT showed a slight decrease in ZOI (9.22 ± 0.8 mm), marketed cream also showed lesser ZOI (11.84 ± 1.2 mm). But the BT-NPopG showed significant enhancement in the ZOI (17.35 ± 1.8 mm) at 48 h. The effect of pure BT, marketed cream, and BT-NPopG were also tested against *A. niger* and the zone of inhibition was measured at 24 and 48 h. The pure BT depicted the ZOI of 13.83 ± 0.8, 14.85 ± 0.9, and 16.15 ± 1.3 mm after 24 h of study. There was a slight decrease in ZOI was observed with the pure BT (10.12 ± 1.7 mm) and marketed cream (13.32 ± 2.1 mm). There was a significant enhancement in the ZOI was observed from BT-NPopG, it showed the ZOI (20.54 ± 1.8 mm) at 48 h. There was a significant (*p* < .05) enhancement (1.2-fold) in the ZOI was observed after 48 h from BT-NPopG. The order of antifungal activity among the formulations is shown as follows pure BT < marketed cream < BT-NPopG at both time points. BT-NPopG exhibited a significant (*p* < .001) enhancement in antifungal efficacy than pure BT, marketed cream. The higher activity observed due to various factors like enhanced solubility, nanoparticle size, slow drug release of BT as well as the presence of chitosan in the gel also has some antibacterial activity. BT-NPopG showed strong antifungal activity against the selected organism because the ZOI >20 mm is referred to as a strong antimicrobial agent (according to antimicrobial activity classification ZOI > 20 mm = strong activity) (Lv et al., 2011).

### Stability study

The stability study of BT-NPopG was assessed as per the ICH guidelines at different temperatures 4 °C, 25°/60% RH, and 40 °C/75% RH, and the results are shown in Table 6. It was observed that there were no significant changes (*p* > .05) in the physical appearance (color, consistency, homogeneity) at the tested condition. The drug content, viscosity, and spreadability were found to be within the limit. So, it can be concluded that the prepared formulation is stable in all storage conditions.

**Table 6.** Stability study results of the optimized gel (BT-NPopG) at per ICH guidelines.

Time (days)	Temperature								
	Drug content (%)			Viscosity (cps)			Spreadability		
	4 ± 1 °C	25 ± 1 °C	40 ± 1 °C	4 ± 1 °C	25 ± 1 °C	40 ± 1 °C	4 ± 1 °C	25 ± 1 °C	40 ± 1 °C
0	99.87 ± 0.9	99.21 ± 1.2	99.87 ± 2.1	35,276 ± 14	35,276 ± 14	35,266 ± 11	10.76 ± 1.9	10.96 ± 1.1	10.95 ± 1.1
30	99.17 ± 1.1	98.87 ± 1.4	98.12 ± 1.5	35,296 ± 12	35,271 ± 13	35,249 ± 14	10.72 ± 0.8	10.93 ± 1.4	10.76 ± 1.3
60	98.11 ± 1.4	98.11 ± 0.8	98.01 ± 1.3	35,316 ± 10	35,256 ± 15	35,238 ± 14	10.66 ± 1.2	10.92 ± 0.8	10.71 ± 0.8
90	98.02 ± 1.2	97.71 ± 1.5	97.66 ± 1.1	35,282 ± 20	35,242 ± 15	35,233 ± 13	10.71 ± 1.3	10.89 ± 1.1	10.62 ± 0.9

## Conclusion

BT loaded PLGA nanoparticles were prepared by the emulsification -sonication method. The formulation was further optimized by Box Behnken design using PLGA (A), PVA (B), and sonication time (C) as formulation factors. Their individual, as well as combined effects, were observed on particle size and entrapment efficiency. Further, the optimized BT-NPopG was converted to gel using chitosan as a mucoadhesive polymer. The characterization of gel formulation revealed optimum pH, spreadability, viscosity, and drug content. The sustained drug release was achieved with both BT-NPop and BT-NPopG. The permeation study result showed enhanced BT permeation and retention from BT-NPopG than BT-NPop, make them ideal topical delivery. The irritation study and antifungal study depicted that the prepared formulation was found to be non-irritant as well as it showed better antifungal activity.
